# Fostering Sound Mind and Communication among Medical Students: Response to the Letter to the Editor

**DOI:** 10.31662/jmaj.2022-0005

**Published:** 2022-02-28

**Authors:** Soichiro Saeki, Masako Shimato

**Affiliations:** 1 Faculty of Medicine, Medical School, Osaka University, Osaka, Japan; 2 Department of Global and Innovative Medicine, Graduate School of Medicine, Osaka University, Osaka, Japan; 3 UCL Medical School, Faculty of Medical Sciences, University College London, London, United Kingdom

**Keywords:** Medical Students, Burnouts, Clinical Clerkship, Imposter Syndrome, COVID-19

We thank Al-Hussainy et al. ^(1)^ of the University of Cambridge for their interest in our previous manuscript in the *Journal* entitled “Mental Health Support for the Current and Future Medical Professionals during Pandemics” ^(2)^. In their letter, the authors have shared their insight through their experience in clinical placements and offered constructive discussions with respect to the impaired clinical exposure and the feelings of imposter syndrome among medical students.

We are in full consensus with the authors ^(1)^, to highlight the impact of the pandemic pushing medical students into imposter syndrome. Although regional differences can be seen among the prevalence of imposter syndrome among medical students and training physicians in prepandemic studies ^(3)^, its importance should be addressed at a global level to establish better support systems to be accessible for all medical students and professionals. Studies on imposter syndrome comparing the situations before and after the pandemic, as well as including regions which have not yet been investigated are now in great necessity.

Furthermore, we are delighted to hear from other medical students who have gone through a similar hardship during the COVID-19 pandemic ^(1)^. The authors have provided their insight on organizing both reflection and practical skill teaching to support mental well-being and clinical competence of medical students. Similar opinions have also been reported from medical students in Singapore ^(4)^, and we believe that such good practice should be shared across the entire medical education community.

In addition, we would like to invite colleagues in medical schools all around the world to share their experiences related to COVID-19. We believe that there are insights on medical education that only students can propose, as they are the ones who faced and had to adjust to radical changes in their education. Their opinions would be of great importance in the future, particularly during worldwide natural disasters, such as new pandemics. We are especially alarmed that reports from Japan have been scarce ^(5)^, and hope that our discussions in this *Journal* would encourage other fellow students to share their perspectives on our discussion.

The letter from Al-Hussainy et al. ^(1)^ supports the classic lesson that local practice can lead to global practice, especially in health promotion. As the restrictions due to the pandemic limited in-person networking both at local and international levels, we are grateful that the *Journal* enables us to start a new dialogue with colleagues worldwide. Although we may have been physically “confined,” our perspectives and constructive opinions must “break free,” in order to collaboratively develop more resistant and secure healthcare systems across the globe.

## Article Information

### Conflicts of Interest

None

### Acknowledgement

SS is supported by the Osaka University Medical Doctor Scientist Training Program.

### Author Contributions

SS conceptualized the basic concept and MS critically revised the contents of the primary manuscript. Both authors edited the primary manuscript, read and approved the final manuscript.

### Approval by Institutional Review Board (IRB)

Not applicable
